# Can cereal-legume intercrop systems contribute to household nutrition in semi-arid environments: A systematic review and meta-analysis

**DOI:** 10.3389/fnut.2023.1060246

**Published:** 2023-01-26

**Authors:** Vimbayi Grace Petrova Chimonyo, Laurencia Govender, Melvin Nyathi, Pauline Franka Denise Scheelbeek, Dennis Junior Choruma, Maysoun Mustafa, Festo Massawe, Rob Slotow, Albert Thembinkosi Modi, Tafadzwanashe Mabhaudhi

**Affiliations:** ^1^Centre for Transformative Agricultural and Food Systems (CTAFS), School of Agricultural, Earth and Environmental Sciences, University of KwaZulu-Natal, Pietermaritzburg, South Africa; ^2^International Maize and Wheat Improvement Center (CIMMYT)-Zimbabwe, Harare, Zimbabwe; ^3^Centre for Transformative Agricultural and Food Systems (CTAFS), Dietetics and Human Nutrition, School of Agricultural, Earth and Environmental Sciences, University of KwaZulu-Natal, Pietermaritzburg, South Africa; ^4^Agricultural Research Council, Vegetables and Ornamental Plants (ARC-VOP), Pretoria, South Africa; ^5^Centre on Climate Change and Planetary Health, London School of Hygiene and Tropical Medicine, London, United Kingdom; ^6^Future Food Beacon Malaysia, School of Biosciences, University of Nottingham Malaysia, Semenyih, Selangor, Malaysia; ^7^Centre for Transformative Agricultural and Food Systems (CTAFS), School of Life Sciences, University of KwaZulu-Natal, Pietermaritzburg, South Africa; ^8^International Water Management Institute (IWMI), Pretoria, South Africa

**Keywords:** cereal–legume intercropping, multicrop agriculture, nutrient dense food, SDG #2, water use efficiency, nutritional water productivity

## Abstract

**Introduction:**

Intercropping cereals with legumes can intensify rainfed cereal monocropping for improved household food and nutritional security. However, there is scant literature confirming the associated nutritional benefits.

**Methodology:**

A systematic review and meta-analysis of nutritional water productivity (NWP) and nutrient contribution (NC) of selected cereal-legume intercrop systems was conducted through literature searches in Scopus, Web of Science and ScienceDirect databases. After the assessment, only nine articles written in English that were field experiments comprising grain cereal and legume intercrop systems were retained. Using the R statistical software (version 3.6.0), paired *t*-tests were used to determine if differences existed between the intercrop system and the corresponding cereal monocrop for yield (Y), water productivity (WP), NC, and NWP.

**Results:**

The intercropped cereal or legume yield was 10 to 35% lower than that for the corresponding monocrop system. In most instances, intercropping cereals with legumes improved NY, NWP, and NC due to their added nutrients. Substantial improvements were observed for calcium (Ca), where NY, NWP, and NC improved by 658, 82, and 256%, respectively.

**Discussion:**

Results showed that cereal-legume intercrop systems could improve nutrient yield in water-limited environments. Promoting cereal- legume intercrops that feature nutrient-dense legume component crops could contribute toward addressing the SDGs of Zero Hunger (SDG 3), Good Health and Well-3 (SDG 2) and Responsible consumption and production (SDG 12).

## Introduction

Sustainable intensification (SI) in agriculture has been part of a multipronged approach that seeks to optimize efficiencies and reduce losses within crop production systems (1, 2). Demand for agriculture that supports a healthier diet is less dependent on monocultural systems and external inputs and is better suited to marginal and semi-arid environments has revived interest in diverse traditional systems (3). Notably, intercropping is recognized as a viable traditional SI technique within semi-arid regions with the potential to improve household food and nutrition security while minimizing the negative impacts of continuous cereal monocropping (4, 5). Previous research has shown that unhealthy diets, such as those high in sugar and sodium, are associated with diseases such as type 2 diabetes (6, 7) and that the food we eat can have environmental impacts such as excessive use of water and climate disruption (8).

Cereal-legume intercrop systems are particularly beneficial in marginal sub-Saharan African (SSA) landscapes, which are characterized by high levels of malnutrition, resource limitation and rainfall variability. These conditions are further exacerbated by climate-related risks and uncertainty (9). Apart from enhancing water and nutrient use efficiency, improving soil fertility (9, 10), and financial gains (11, 12), cereal-legume intercrop systems have become a better bet for increased food and nutrition security in marginal farming communities (13). However, concerns have arisen on whether intercropping can move food security beyond calories produced per capita and address household nutritional needs (4, 14–16).

Within the study of intercropping, increases in “food security” are usually extrapolated from any positive gains in productivity (17), and improvement in “nutritional security” is assumed as a result of increased crop diversity (18, 19). However, the latter may not always be accurate, especially for household nutritional contributions within marginal farming communities. From a nutritional point of view, mineral bioavailability from cereals and legumes is usually low due to their high dietary fiber content or the presence of antinutritional components like phytic acid, oxalate, or polyphenols that may interfere with mineral absorption (20). Antinutritional components further confound the purported nutrient gains within cereal-legume intercrop systems. Furthermore, under increased water stress conditions such as drought, antinutritional factors have been found to increase, decreasing the overall nutritional quality of a crop (21). Depending on species, growing conditions (e.g., water and fertilizer) and cooking method, the bioavailability of minerals in legumes range between 5 and 35% (22), while for cereal crops, it ranges between 20 and 80% (23, 24). There is, therefore, a need to understand the impacts of water on yield and nutritional yield, as this impacts household food and nutrition security, especially in water-stressed environments.

Nutritional water productivity (NWP) is an emerging concept that combines information on the nutritional value of crops with that of crop water productivity. Here, crop Water productivity is defined as the economic or biophysical gain from using a unit of water consumed in crop production. The combination of a crop’s nutritional value and crop water productivity makes NWP a useful index for evaluating the impacts of agriculture on food and nutrition security, especially under limited water availability (25). The NWP index provides a way of understanding the complex and dynamic interlinkages between crop water use and the nutrient value of a crop or cropping system. The NWP index also allows for a holistic assessment of water, food, and nutrition security (25–28). What is desirable is a higher NWP, which means more nutrients for less water. The quantification of NWP within agricultural systems is in its infancy, with (26) evaluating NWP for legumes [bambara groundnut (*Vigna subterranea*), cowpea (*Vigna unguiculata*), groundnut, (*Arachis hypogaea*) and dry bean (*Phaseolus vulgaris*)], and (25) in leafy vegetables [amaranth (*Amaranthus cruentus*) spider flower (*Cleome gynandra*), Swiss chard (*Beta vulgaris*)] and orange-fleshed sweet potatoes (*Ipomoea batatas*). Presently, this is only one of a few studies that have evaluated the NWP of multi-crop systems.

We hypothesized that cereal-legume intercropping could improve household nutritional contribution through the improvements in NY, WP, and NWP compared to cereal monocropping. Therefore, this study aimed to synthesize and analyses available published evidence on the NWP of cereal-legume intercrop systems and, in addition to that, their potential to improve the sustainability of food systems in water-scarce environments. It should be noted that the work presented is exploratory.

## Methodology

### Identification of studies

**FIGURE 1:**
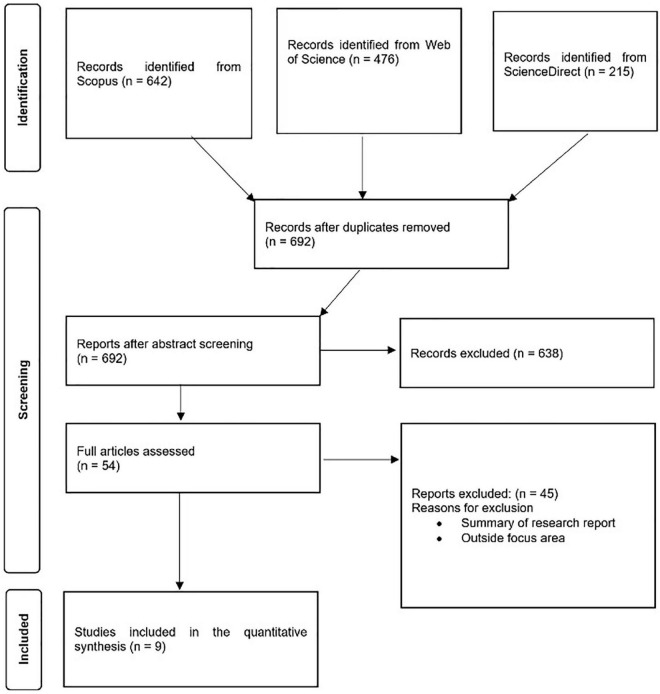
PRISMA flow diagram used for selection of studies.

Three databases (Scopus, Web of Science and ScienceDirect) were used to search for published peer-reviewed literature on cereal–legume intercrop systems from 1980–2022, using the PRISMA methodology (Figure 1). The search terms used were (“intercrop*” OR “mixture*” OR “multicrop*”) AND (“water use*” OR “water use efficiency” OR “water productivity”) and (“Kidney bean” OR “Common bean” OR “Lima bean” OR “Adzuki bean” OR “Mung bean” OR “Black gram” OR “Scarlet runner bean” OR “Ricebean” OR “Moth bean” OR “Tepary bean” OR “Horse bean” OR “Broad bean” OR “Field bean” OR “Garden pea” OR “Pea” OR “Protein pea” OR “Chickpea” OR “Cowpea” OR “Pigeon pea” OR “Lentil” OR “Bambara groundnut” OR “Vetch” OR “Lupins” OR “Lablab” OR “Jack bean” OR “sword bean” OR “Winged bean” OR “Velvet bean” OR “Yam bean”) and (maize OR rice OR wheat OR durum OR barley OR sorghum OR millets OR oats OR triticale OR rye OR fonio OR teff OR “Wild rice” OR spelt OR einkorn OR emmer OR kamut OR “Canary grass” OR quinoa OR amaranth OR buckwheat OR kaniwa OR pitseed OR goosefoot).

### Inclusion and exclusion criteria

The initial search retrieved 1,333 articles; after that, articles were screened for duplicates, and 692 remained. We retained articles written in English and excluded those not written in English. From the remaining 662 articles, titles and abstracts were examined to check whether studies were field experiments comprising grain cereal and legume intercrop systems. We excluded all articles not considering grain cereal and legume intercrop systems. Following the screening, 54 abstracts remained. Full-length articles were only considered if they measured plant parameters included yield (Y) and water use {WU -[evapotranspiration (mm), rainfall (mm) received and/or water used (m^3^)]} and the experiments presented yield (in kg ha^–1^, t ha^–1^, and g m^–2^) values for monocrop treatments for both intercrop component crops. This was to allow yield and nutrient yield comparisons between monocrops and intercrops to be compared. Articles that did not meet these criteria were excluded. Overall, 9 articles met the inclusion criteria (refer to Supplementary Information 1, Table 1 for the PRISMA flowchart). Since the work was largely exploratory and, given the limited number of retained articles, we did not consider bias. A glossary of key terms has been provided in Supplementary Information 2.

### Data extraction

Multiple data records were extracted from each publication based on the number of experiments and appropriate treatments within experiments in an article. We extracted site-specific data (geographic coordinates and mean annual rainfall (mm) (Supplementary Information 1, Table 2), management data (plant densities, fertilizer and water management), yield, water applied as well as water productivity (Supplementary Information 1, Table 3). Data were directly extracted from published tables or digitized graphs using WebPlotDigitizer (29). All grain yield data were presented as t ha^–1^. Only treatment mean values were extracted regardless of the number of replications.

#### Nutrient content

A second literature search on nutrient concentration (NC) of crops was performed to quantify the nutrient yield (NY) for each system (cereal monocrop and intercrop). We focused on estimating the NY of three essential micronutrients, Fe, Zn, and Ca, within the intercrops (30). This selection reflects some of the micronutrients of public health interest because of either existing widespread deficiency (Fe and Zn) or because Ca intakes are low in developing countries (31). We also included carbohydrates, fiber, and protein as essential macronutrients. A detailed description of why the selected nutrients were included in this study is presented in Supplementary Information 3.

Data on proximate and nutrient composition for crop species were sourced from databases such as the United States Department of Agriculture Food Composition^1^ and the Food and Agricultural Composition/In Foods.^2^ We also used peer-reviewed literature obtained from the above-mentioned electronic databases. The search terms were “proximate composition,” “nutrient composition,” “nutrient yield,” “cereal,” “legume,” “maize,” “sorghum,”’ “pearl millet,” “wheat,” “dry bean,” “soybean,” “cowpea,” “groundnut,” “pea,” “chickpea” (refer to Supplementary Information 1, Table 4 for the search strings used). Similar to the intercrop system data, the database comprised articles published in English between 1980 and 2019. The selection criterion was that proximate compositions and nutrient concentrations must be reported in nutrient concentration per 100 g (g mg^–1^ per 100 g). After screening, eight articles and ten nutrient composition databases from usad.gov and fao.org were used to develop the proximate composition and nutrient content data to calculate NWP for the crops included in the study. The average grain nutrient composition (per 100 g at 12 % moisture) of cereals and legumes pooled from the literature on the identified crops can be found in Supplementary Information 1, Table 4.

To calculate available nutrients, we assumed a modest value of 70% of legume nutrients are unavailable for absorption due to limited bioavailability inside human bodies (22, 23, 32). We assumed that around 35% are unavailable for absorption for cereal crops due to limited bioavailability inside human bodies (23, 25). Using the method outlined by Nyathi et al. (25), the percentage contribution of an intercrop system, after adjusting for bioavailability, to the daily recommended nutrient intake was calculated according to Kruger et al. (33). Refer to Supplementary Information 1 and Tables 4, 5 for calculations of the bioavailability of nutrients.

### Nutrient yield (NY), nutritional water productivity (NWP) and potential contribution to human nutrition

The nutrient yield of intercrop systems was calculated based on an equation adapted from (34) as follows


(1) $$NY_{s}=\left(Y_{I\,C}\times \%NC_{C}\right)+\left(Y_{I\,L}\times \%NC_{L}\right)$$

(2) $$NY_{c}=\left(Y_{C}\times \%NC_{C}\right)$$

where *NY*_*C*_ and *NY*_*s*_ are the nutrient yield for cereal (C) in the monocrop system and corresponding intercrop system (S), *Y*_*C*_ is the grain yield of the cereal in the monocrop system (C), *Y*_*IC*_ is the grain yield of the cereal component (C) in intercrop system (S), *Y*_*IL*_ is the grain yield of the legume component L in intercrop system S, *NC*_*C*_ is the nutrient concentration of the cereal, and *NC*_*L*_ is the nutrient concentration of the legume component.

Nutritional water productivity (NWP) of intercrop systems was calculated as a ratio based on a formula adapted from (35):


(3) $$N\,W\,P_{C/S}\,\left(k\,g\,m^{-3}\right)=W\,P_{C/S}\,\left(k\,g\,m^{-3}\right)\times N\,C_{C/S}\,g\,100\,g^{-1}$$

where NWP of cereal monocrop (C) or intercrop (S) system is the nutritional water productivity, WP of cereal (C) and intercrop (S) system is defined as the amount of agricultural output produced per unit of water used. Water productivity (25) for monocrop and intercrop systems was calculated as


(4) $$W\,P_{C}=\frac{Y_{c}\,\left(k\,g\,h\,a^{-1}\right)}{w\,a\,t\,e\,r\,u\,s\,e\,d\,\left(m^{-3}\right)}$$

(5) $$W\,P_{S}=\frac{Y_{I\,C}\,\left(k\,g\,h\,a^{-1}\right)}{w\,a\,t\,e\,r\,u\,s\,e\,d\,\left(m^{-3}\right)}+\frac{Y_{I\,L}\,\left(k\,g\,h\,a^{-1}\right)}{w\,a\,t\,e\,r\,u\,s\,e\,d\,\left(m^{-3}\right)}$$

#### Household nutritional contribution

We determined the probability that average households could meet their nutritional needs by growing the cropping systems examined in this study. Nutrients obtained from foods consumed vary depending on the portion size consumed, amount of food utilized, and food preparation and processing (36). To estimate the nutritional requirements for a household of four individuals [Male and female adults, an adolescent female and a child (4–8 years old)], the Estimated Average Requirement (EAR) was calculated from the Dietary Reference Intake (DRI) (Supplementary Information 1, Table 6). The EAR value is an estimated daily value for a specific nutrient that meets 50% of a specific age group and gender. It is seen as a primary reference for assessing nutrient adequacy. Estimated weight was used for each household member to determine the protein requirements. Uusiku et al. (37) used a similar methodology; however, our results for nutritional requirements differ. One of the reasons for this difference is that Uusiku et al. (37) used the recommended Daily Nutrient Intakes (RNIs), and this study used the DRI values, which have replaced and expanded on the RNIs and Recommended Dietary Allowances (RDA) (38).

The per cent contribution to EAR was calculated as


%contribution[Nutrientconcentration(gormg 100g-1)/

(6) $$Nutrientconcentration\left(gormgday^{-1}\right)]\times 100$$

### Data visualization and statistical analyses

Publication bias is a challenge in meta-analysis. This bias often occurs when the published studies report larger or more significant effect sizes (e.g., the effect of a treatment). Also, published studies are more likely to have found significant and/or larger yield changes than unpublished studies. Our study acknowledges that if any publication bias was present, it impacted the results of this meta-analysis since we used the resultant yield data from each selected publication. However, due to the limited number of articles identified, we did not subject the data to any statistical correction. Using the R statistical software (version 3.6.0) (39), paired *t*-tests were used to detect the difference between the sole cereal and intercropped cereal. We also used generalized linear mixed analysis (GLMM) at 95% confidence levels to determine if differences existed between the intercrop system and the corresponding cereal monocrop for Y, WP, NC, and NWP. Descriptive statistics such as means, standard deviations, bubble chart box and whisker plots were used to analyses outputs. Box and whisker plots can show stability and general distribution of the data sets. The bar charts visualized the relationship between two or more variables and helped assess co-dependent variables. On the other hand, box and whisker plots can show stability and general distribution of data.

## Results and discussion

### Literature review

The intercrop data consisted of nine articles (Supplementary Information 1 and Tables 1, 2) representing five countries from two continents. The locations were China (3 articles), South Africa (2), Kenya (1), Ethiopia (1), and India (1). The cereal component crops included maize (*Zea mays* L.), pearl millet (*Pennisetum glaucum*), sorghum (*Sorghum bicolor*) and wheat (*Triticum aestivum*). At the same time, the legumes intercropped were dry beans (*P. vulgaris*), cowpea (*Vigna unguiculata*), pea (*Pisum sativum*), groundnut (*Arachis hypogea*), and chickpea (*Cicer arietinum*). The various crop species identified highlighted the potential of intercropping in improving crop diversity. Crop diversity is critical in food and nutrition security (40) and dietary diversity (41). Maize intercrop systems were dominant (five out of eight); this is consistent with the global importance of maize as a staple crop (40). Also, water use issues are more prudent in maize production systems due to the crops’ higher requirements than the other cereal crops (42–45). Most intercrop systems (six out of eight) were grown in semi-arid regions (average 550 mm annual rainfall), indicating the potential of intercrop systems to do well under conditions of low water availability. However, the limited number of articles found during the literature search highlights the need for more research focusing on WU and NY in intercrop systems.

### Yield, land equivalent ratio, and water productivity

Table 1 presents the grain yield (Y) of selected cereal crops and legumes (monocropping and intercropping) reported in the selected studies. The mean values for the cereal monocrops (*n* = 23 experiments) ranged from 0.9 to 11.0 t ha^–1^; the highest and lowest cereal Y was observed from maize and sorghum, respectively. For the intercrop, the mean values for cereal Y ranged from 0.8 to 8.9 t ha^–1^; similarly, maize and sorghum exhibited the highest and lowest Y, respectively. The mean Y for legume monocrops ranged from 0.1 to 3.9 t ha^–1^, whereas for the intercrop system, it ranged from 0.1 to 2.1 t ha^–1^ (Table 1). Groundnut and cowpea exhibited the highest and lowest mean Y for monocrop and intercrop systems, respectively. The differences in species mean Y could be attributed to differences in Y potentials for each crop species (46, 47). Compared to maize, groundnut and soybean, commercial crops with high Y potential, millet, sorghum and cowpea, are regarded as underutilized crop species with limited crop improvement and low yield potential (48).

**TABLE 1:** Yield and water productivity ranges for monocrop and intercropped systems across reviewed studies.

Cropping system		Cereal yield (t ha^–1^)		Legume yield (t ha^–1^)		Land equivalent ratio	Water productivity^2^ of monocrop systems (kg m^–3^)		Water productivity of intercrop systems (kg m^–3^)
Cereal	Legume	Monocrop	Intercrop	Monocrop	Intercrop		Cereal	Legume	Cereal
Maize	Cowpea	0.4–1.5 (0.9)^1^	0.2–1.5 (0.8)	0.4–0.6 (0.5)	0.1–0.6 (0.3)	0.2–2.1 (1.2)	0.25–0.82 (0.56)	0.18–0.38 (0.27)	0.1–1.00 (0.56)
	Dry bean	2.5–10 (7.0)	1.3–9.0 (6.0)	1.7–5.4 (3.5)	0.4–1.9 (1.1)	1.1–1.6 (1.2)	0.86–1.33 (1.08)	0.31–0.68 (0.56)	0.79–1.35 (1.09)
	Pea	6.7–17.4 (11)	6.1–12.7 (8.9)	1.6–1.9 (1.7)	0.8–1.8 (1.4)	0.6–2.4 (1.6)	1.09–3.07 (1.85)	5.5–6.8 (6.1)	1.51–2.38 (1.90)
	Groundnut	4.0	2.4–3.7 (3.2)	1.8	0.4–1.3 (0.7)	1.1–1.4 (1.3)	0.47	0.21	0.43–0.51 (0.47)
	Soybean	4.0	2.3–3.9 (3.3)	1.5	0.9–1.4 (1.2)	1.1–1.5 (1.4)	0.47	0.17	0.45–0.55 (0.49)
Fox millet	Groundnut	4.8 - 5.4 (5.1)	2.6 - 3.9 (3.3)	3.7 - 4.2 (3.9)	1.6 - 2.7 (2.1)	0.9–1.4 (1.2)	1.21–1.45 (1.32)	0.10–0.12 (0.11)	1.26–1.93 (1.47)
Sorghum	Cowpea	0.8–1.2 (0.9)	0.8–0.4 (1.0)	0.0–0.2 (0.08)	0.0–0.1 (0.05)	1.1–1.9 (1.2)	0.24–0.59 (0.40)	0.04–0.10 (0.08)	0.20–0.59 (0.37)
Wheat	Chickpea	1.5–4.3 (3.0)	1.1–2.6 (1.9)	1.7–7.3 (3.8)	0.5–2.4 (1.1)	0.9–1.0 (0.9)	0.70–1.42 (1.02)	0.18–0.46 (0.22)	0.56–1.21 (0.96)
Grand mean						1.3 (± 0.6)^2^	0.98 (± 0.76)^2^	0.38 (± 0.11)^2^	1.20 (± 0.41)^2^

^1^Number in brackets represents the mean; ^2^Standard deviation.

On average, intercrop systems produced 14% [95% CI 0–4.5 t ha^–1^] lower cereal Y than monocrop Y. The most considerable yield differences when comparing intercropping to monocropping were observed from the fox millet–groundnut (−53−20%), maize-dry bean (−35 to −10%), and wheat-chickpea (−32 to −12%) systems. The low Y observed for intercropped cereal and legumes relative to their monocrop systems could be attributed to increased resource competition between the intercropped crops. In multicrop systems, competition between component crops is always present as the crops require the same growth resources, e.g., water, nutrients, and solar radiation (4, 49). The competition for these resources is significantly increased when critical growth stages such as canopy expansion, flowering and grain filling overlap, as could have been the case for the aforementioned systems (50). Then again, where there are lower yield reductions, it could be that the cereal and legume crops are complementary. Chimonyo et al. (4) and Smith et al. (15) observed complementary interactions for the maize-pigeon pea intercrop system, which was attributed to asynchronous phenology and less competition for growth resources during critical growth stages. The low Y in the maize–cowpea system was consistent with low plant populations, nitrogen fertilizer rates, and, more importantly, low water availability (314 mm; Supplementary Information 1, Table 2). To reduce yield gaps in cereal–legume systems, there is a need to exploit crop interactions to manage competition between cereals and legumes. Under low water availability, manipulating agronomic practices such as sowing time, sowing density, and N fertilizer rate can enhance species complementarity and total productivity, optimizing productivity (50).

Water productivity for cereal monocrops ranged from 0.40 to 1.85 kg m^–3^, whereas for legume monocrops, it ranged from 0.08 to 0.61 kg m^–3^. The differences in WP between the cereal and legume crops can be attributed to differences in physiology (51). Several studies have shown that C_4_ plants are more efficient at carbon fixation and have a higher WP than C_3_ plants owing to photosynthetic pathways (52–54). This could explain why many of the C_4_ cereals showcased in the study had higher WP than C_3_ cereals and legumes. The differences in WP between the cereal or legume crops could also be attributed to the differences in Y potential across the different crop species (46, 47). The study results highlighted that intercropping (0.47 to 1.90 kg m^–3^) improved WP by 19% [95% CI 0.0–0.44 kg m^–3^] compared to monocropping. It could be assumed that improved WP could be due to increased capture and use of unproductive water (water that is not taken up and transpired by the plant and is lost to plant production through deep drainage or evaporation) (55). The wide range for WP was consistent with the observed variation in Y (correlation = 0.81, *P* < 0.05). Maize–groundnut (0.47 kg m^–3^), maize – soybean (0.49 kg m^–3^), and sorghum–cowpea (0.37 kg m^–3^) intercrop systems showed the lowest WP, while maize–pea (1.90 kg m^–3^) and millet–groundnut (1.49 kg m^–3^) showed the highest WP (Table 1).

### Nutrient concentrations (NC), nutrient yield (NY), and nutritional water productivity (NWP)

**FIGURE 2:**
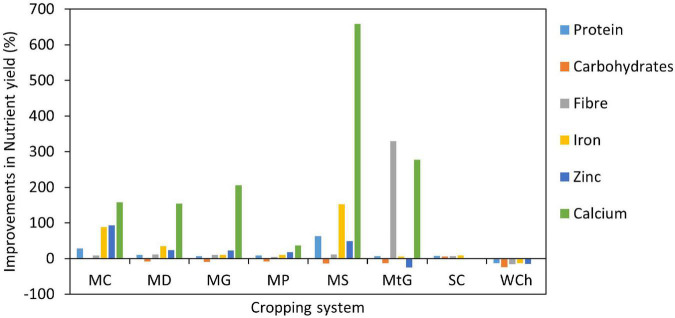
Improvements in nutrient yield (NY) for Carbohydrates, Protein, Fiber, Iron, Zinc, and Calcium across the intercrop systems relative to monocropping. The intercrop systems used in the study include Maize-Cowpea (MC), Maize-Dry Bean (MD), Maize-Groundnut (MG), Maize-Pea (MP), Mazie-Soybean (MS), Millet-Groundnut (MG), Sorghum-Cowpea (SC), and Wheat-Chickpea (WC).

We calculated the nutrients that can be harvested per unit area of land (NY) for selected cereals and legumes (monocropping and intercropping) (Supplementary Information 1). Excluding the results of carbohydrate Y, this study indicated that intercrop systems had higher NY than corresponding cereal monocrops. Intercropping improved fiber, Fe and Ca Y by 10, 15, and 135%, respectively (Figure 2). The most substantial improvements in Ca Y were observed in the maize-soybean intercrop system, with a 658% increase compared to the corresponding maize monocrop (Figure 2). The maize-soybean intercrop system also significantly improved protein (63%) and Fe (152%). The improvements can be attributed to the legumes used within the intercrop systems. For instance, soybean was seen to have a comparative advantage over other legumes as they contain a more significant amount of protein, Ca and Fe (56, 57). In this regard, legumes could be considered a good, cheaper alternative to animal protein and Ca (58) and can improve the nutritional value of starch-based foods despite low bioavailability. When a starch-based food is consumed with legumes, it provides other complementary proteins (59) and enhances Ca absorption (60). Starch-based foods lack lysine and tryptophan found in legumes, and the sculpture-containing amino acids limiting in legumes are found in starch-based foods (61). It could be assumed that the protein quality of a starch-based cereal could be improved when consumed with a legume, thus, contributing to the reduction of protein-energy malnutrition (62–64). It is recommended that the assessment of protein content in foods, such as legumes, should be measured by the sum of individual amino acids (61, 65). However, these data are not always readily available, and it is acceptable to estimate protein content based on total nitrogen content (66). This study showed that intercropping cereals with legumes did not always improve Zn contributions. While legumes contain more Zn than cereal crops, the bioavailability and absorption are affected by the total protein content within the legume and phytic acid found in several cereal crops (67). The consumption of cereal and legumes with Zn-rich vegetables should be encouraged.

**FIGURE 3:**
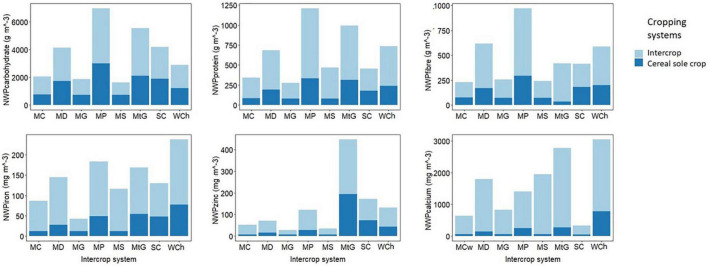
A comparison between intercrop systems and corresponding cereal monocrop for nutritional water productivity (NWP) for Carbohydrates, Protein, Fiber, Iron, Zinc, and Calcium. The intercrop systems used in the study include Maize-Cowpea (MC), Maize-Dry Bean (MD), Maize-Groundnut (MG), Maize-Pea (MP), Mazie-Soybean (MS), Millet-Groundnut (MG), Sorghum-Cowpea (SC), and Wheat-Chickpea (WC).

The results indicated that carbohydrates’ nutritional water productivity (NWP) values were moderately higher under intercropping than monocropping (Figure 3 and Supplementary Information 1, Table 8). The values ranged from 238 to 5,047 g m^–3^ for intercrop systems, whereas for monocropping, the values ranged from 258 to 4,888 g m^–3^. The increase in carbohydrate NWP and the NWP for all the nutrients under investigation agreed with our hypothesis that intercropping cereals with legumes would improve NY. From a water perspective, the results suggest that improving NWP might require less water to produce comparable yields in intercropping to those produced in cereal monocrops. However, when considering human nutrition and health, an increase in carbohydrate NWP could be detrimental to poor rural households that consume a high-energy diet. As suggested by our results, the legume component’s benefits are that any carbohydrate content reductions resulting from a cereal yield reduction are offset by legume carbohydrate content. In addition, there is a bonus of the addition of other nutrients. When comparing the results of monocropping and intercropping, there was an overall improvement in NWP for protein, fiber, Fe, Zn and Ca by 55, 52, 60,45, and 82%, respectively (Figure 2). Under low water availability and within cereal–legume intercrop systems, the slight improvement in carbohydrate yield can be considered a worthwhile trade-off for an overall improvement in NY. This improved balance between nutrients, especially protein and carbohydrates, can reduce protein-energy malnutrition and obesity–under and over-nutrition, respectively.

The most considerable improvement for NWP was for Ca, which showed an 82% improvement under intercropping relative to cereal monocrops (Figure 2). For Ca-NWP, intercropping (32 to 3,287 mg m^–3^) indicated higher values than monocropping (21 to 945 mg m^–3^). The improvements in Ca-NWP were associated with the legume species used in the systems. A closer look at maize systems showed that the highest Ca-NWP improvement was when soybean was used, while the least improvements were observed when peas were used. Soybean has high Ca content that averages 35.7 mg 100g^–1^ compared to other legumes such as pea (4.9) and groundnut (19.3). As a commercially important food and feed crop, soybean has undergone significant genetic improvements for improved nutrient yield (68). Although soybean is not, particularly drought tolerant, intercropping it with cereals under water-scarce conditions could be viable for improving the overall system Ca NWP.

### Household nutritional contribution

**FIGURE 4:**
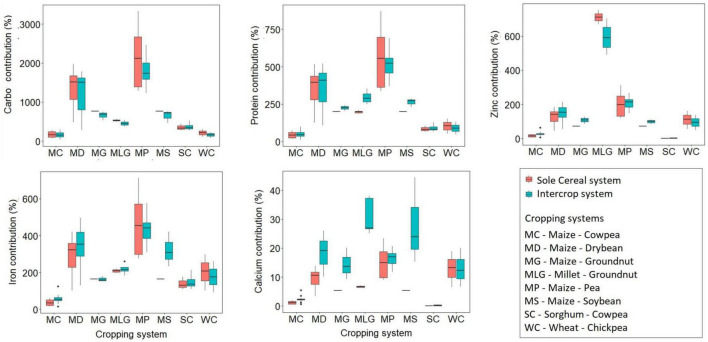
A comparison between intercrop systems and corresponding cereal monocrop for Nutrient Contribution (NC) in terms of Estimated Average Requirement (EAR) per cent for each nutrient for an average family of four comprising an adult male and female, an adolescent female, and a child. Carbo is the abbreviation for carbohydrates.

The estimated percentage contribution of a cropping system (cereal monocrop and intercrop) for a family of four (comprising of a male and female adult, a female adolescent and a toddler) is presented in Figure 4 and Supplementary Information 1, Table 9. An ideal cropping system for optimum nutritional benefits provided more than 100% estimated average requirement (EAR for a family of four for a year). Maize–pea and millet–groundnut systems and their corresponding maize monocrop systems provided more than 100% of the EAR for a family of four for carbohydrates, protein, Fe, and Zn. Our results illustrated that despite intercropping improving Ca yields, the improvements were inadequate to meet the EAR for a family of four. It was interesting to note that maize-pea systems could provide ten four-member families with EAR for carbohydrates for a year. Millet–groundnut systems produced the highest nutrient contribution of Zn. Most intercrop systems reduce the EAR ratio between carbohydrates and other nutrients. For example, the maize–soybean intercrop system had the lowest carbohydrates to Fe ratio (1: 0.7), millet–groundnut had the lowest carbohydrates to Zn ratio (1:1.5), and wheat–groundnut had the lowest carbohydrates to Ca to protein ratio (1:0.5:0.1). The result shows that different crop species combinations result in different contributions to household nutrition. This would suggest that more than one type of legume species or adding other crop species could improve the balance of NC for a household across the nutrients relative to carbohydrates.

## Study limitations

The number of studies included in this review was small (*N* = 9), limiting our findings’ generalisability. While the number of studies is sufficient for quantitative analysis (69), it should be recognized that it limits the strength of our conclusions. The limited number of studies included was restricted by the search criteria imposed by our research objective. Higgins (70) proposed that a minimum of 10 studies are necessary for conducting analyses. However, Valentine et al. (69) concluded that two studies are adequate for quantitative analysis of secondary data provided that the random and fixed effects are well defined and the effect size of both studies are similar; the current study satisfied these conditions stipulated by Valentine et al. (69).

We obtained Y and WU values from different literature sources to compute water productivity. To standardize the calculation of WP, this paper used total water applied and/or total irrigation amount as the denominator. According to Nyathi et al. (25), the use of transpiration as the denominator, as opposed to water applied or water used, as was used in this study, could provide a more accurate determination of WP. van Halsema and Vincent (71) emphasized the need to use the beneficial use of water (transpiration rather than evapotranspiration) when assessing the water productivity of crops. However, because it is challenging to separate evaporation from evapotranspiration, evapotranspiration is used as a denominator when computing water productivity. Also, when assessing the NWP of the cereal-legume intercropping system, we computed NWP as a product of WP and NC. These values are complex as; (i) they are obtained from different locations, (ii) different management strategies, including soil fertility levels and irrigation water regimes, (iii) different soil types due to different locations, and (iv) different crop species might consist different nutrient concentrations. These factors could influence crop WU and perhaps NC, and ultimately NWP.

## Study implications

Notwithstanding these limitations, our findings show that intercropping cereals with legumes is more beneficial than monocropping in terms of increasing food diversity and nutrient productivity per unit of water and land used. The main findings and implications are summarized in Table 2. Our results suggest that research on the climate-environment-nutritional-benefits of multicropping is scant and uncoordinated. Research to provide insights into nutrition-sensitive cropping systems in marginal environments must be multidisciplinary with standardized protocols and frameworks to ensure harmonization of methods, data collection tools and data. Further, there is a need to integrate socio-economic and bio-physical factors into the assessment of NWP as they might affect the interpretation of the results (12). Since food and nutrition insecurity is synonymous with poverty (72, 73), future studies should include an econometric assessment to determine the cost-benefit from a household nutrition and water perspective for intercropping cereals with legumes. Also, the use of indicators such as Nutrient-Rich Foods (NRF) (74) or Overall Nutritional Quality (75) and nutritional function diversity (19), which are metrics of nutrient density and value, can be used for crop nutrient profiling. Including such methods and metrics will provide a more holistic assessment of the co-benefits of such systems by informing policy on the value of nutrition-sensitive cropping systems for reducing poverty and malnutrition. This will ensure that there is relevant and scalable data to assess the contribution of cereal-legume intercropping and other multicrop systems to achieving sustainable intensification of agricultural systems and sustainable human health and wellbeing outcomes.

**TABLE 2:** Key findings, implications and recommendations grouped according to benefits for productivity, environment and human nutrition.

Domain	Key findings	Implications	Recommendations
Productivity	● Productivity within the intercrop systems was dependent on crop species and management	● Cereal–legume intercrop systems can be modeled to match socio-economic and bio-physical characteristic	● Several iterations are needed with agronomists, irrigation experts, and nutritionists working together to design cereal–legume intercrop systems for improved health and environmental outcomes ● Econometric assessment needs to be done to determine cost and nutrient-benefits of different cereal–legume intercrop systems ● Agricultural policies need to be based on a better understanding of smallholders’ objectives and constraints
	● Underutilized cereal and legume crop species were low yielding when compared to important commercial ones ● Yield instability for the same species across different intercrop systems ● Intercropping reduces the productivity of component crops but increases overall productivity	● Yield gaps within and across crop systems still exist, and this presents opportunities to further intensify cereal–legume intercrop systems	● Adopt management strategies such as asynchronous planting and shifting plant populations can improve yield and yield stability
Environmental	● Under semi-arid conditions intercropping cereal with legume improved WP	● Under low water availability, intercropping cereals with legumes can be used to mitigate the impacts of water stress and drought on crop productivity	● Under low water availability, intercropping should be recommended as a viable water management strategy
	● Several cereal–legume intercrop combinations were identified in the study	● Legumes can contribute to the ecological intensification of cropping systems ● Crop species diversity within cereal and legumes can contribute to enhancing functional biodiversity ● Crop diversification implies economic diversification ● Increased resilience to shocks ● Cereal–legume intercrop systems can enhance environmental sustainability in terms of water use and nutrient cycles	● Efforts to improve marginal production systems require innovative and inclusive approaches that enable adaptation to the socio-ecological context ● There is a need to map ecosystem services provided by cereal–legume intercrop systems
Human nutrition	● High yields for selected nutrients were observed in cereal – legume intercrop systems relative to corresponding cereal monocrop systems	● Cereal–legume intercrop systems can be used to address specific or collective nutrient deficiencies in marginal production systems	● There is a need to do crop nutrient profiling to determine the full nutritional benefits of cereal–legume intercrop systems
	● Intercropping cereals with legumes o reduced the carbohydrate to protein, Fe, and Ca ratio o reduced NWP for carbohydrate, and protein under cereal–legume intercrop systems o increased NWP for Ca, Fe and Zn	● Under low water availability, intercropping cereals with legumes can improve nutrient balance ● More water is required to maintain carbohydrate and protein yields under cereal–legume intercrop systems	● Cropping systems within marginal communities need to be designed to address multiple objectives, including improving nutrient balance for nutrition ● To improve water availability, there is a need to adopt rainwater harvesting and soil water conservation strategies to enhance soil water capture and storage and minimize unproductive loss of soil water
	● Cereal – legume intercrop systems could not provide EAR for Ca	● Nutritional gaps still exist within cereal–intercrop systems, which creates opportunities to refine agronomic management and crop choices	● There is a need to assess nutritional gaps within different intercrop systems ● Increase crop diversity within the systems to improve the yield of limiting nutrients

Yield and nutritional gaps still exist across intercrop systems. Good agronomy resulted in high NY and NWP. There is a need to develop “better bet” agronomic practices to intensify cereal-legume intercrop systems (Table 2) sustainably. In cases where inputs such as fertilizer are limited, as in much of SSA, farmers can opt to intercrop cereals with legumes that have high nitrogen fixation rates (76). Future studies should generate new experimental data focused on exploring the effects of additional factors such as management practices [asynchronous planting and plant density (50)], climate and edaphic factors on nutrient content, and NWP for a range of cereal-legumes systems (Table 2).

In line with resource use management and as water becomes scarcer, we advocate for the use of water footprint (WF) instead of WP as it can help to inform farmers and policymakers on less water-intensive cropping systems. The WF potentially provides a way of better understanding the complex and dynamic interlinkages between water along the whole food production chain and the nutrient value of the crop or a cropping system (77), which should allow for holistic assessment of direct and indirect challenges faced within and across challenges of water and, food and nutrition insecurity.

## Conclusion

We assessed if cereal–legume intercropping could increase NY, WP, and NWP and thereby improve contributions to household nutrition compared to cereal monocropping. Our findings show that intercropping significantly improved NY and NWP for the most investigated nutrients. While almost all of the studied intercrop systems could provide more than 100% of the EAR for carbohydrates, protein, Zn and Fe for a family of four, they could not meet the required EAR for Ca. Species composition was an important factor determining an intercrop system’s relative Y, NY, NWP, and NC. Using NWP as an index provided insights into the nutritional value of different intercrop systems under semi-arid conditions. Thus, NWP could generate evidence for informing context-specific and nutrition-sensitive policies and strategies that promote sustainable and healthy cropping systems within marginalized communities in water-scarce environments. Promoting cereal-legume intercrops that feature nutrient-dense legume component crops could contribute toward addressing several Sustainable Development Goals related to social and environmental outcomes, specifically, SDGs 2 (Zero hunger), 3 (Good health and wellbeing), and 12 (responsible consumption and production).

## Data availability statement

The original contributions presented in this study are included in this article/Supplementary material, further inquiries can be directed to the corresponding authors.

## Author contributions

VGPC and TM: conceptualisation. VGPC, LG, and MN: methodology, investigation, and data curation and analyses. TM: resources and project administration. VGPC, LG, MN, MM, FM, and TM: writing—original draft preparation. PS, FM, DC, RS, ATM, and TM: writing—review and editing. TM, ATM, and RS: funding acquisition. PS, DC, FM, RS, ATM, and TM: critical review and redrafting. All authors contributed to the article and approved the submitted version.

## Footnotes

1
https://fdc.nal.usda.gov/fdc-app.html

2
http://www.fao.org/infoods/infoods/tables-and-databases/faoinfoods-databases/en/
