# Supplementary material for: Can cereal-legume intercrop systems contribute to household nutrition in semi-arid environments: A systematic review and meta-analysis
Source: Front Nutr. 2023 Jan 26;10:1060246. doi: 10.3389/fnut.2023.1060246 (PMC9923432; doi:10.3389/fnut.2023.1060246)
Supplement: Supplementary file 2 [file Table_1.docx]

**Supplementary Information (SI) 1**

Table 1 Records identified through database search and screening procedure

| (“intercrop*" OR "mixture*" OR “multicrop*”) AND (“water use*” OR “water use efficiency” OR “water productivity”)  and  (“Kidney bean” OR “Common bean” OR “Lima bean” OR “Adzuki bean” OR “Mung bean” OR “Black gram” OR “Scarlet runner bean” OR “Ricebean” OR “Moth bean” OR “Tepary bean” OR “Horse bean” OR “Broad bean” OR “Field bean” OR “Garden pea” OR “Pea” OR “Protein pea” OR “Chickpea” OR “Cowpea” OR “Pigeon pea” OR “Lentil” OR “Bambara groundnut” OR “Vetch” OR “Lupins” OR “Lablab” OR “Jack bean” OR “sword bean” OR “Winged bean” OR “Velvet bean” OR “Yam bean”)  and  (maize OR rice OR wheat OR durum OR barley OR sorghum OR millets OR oats OR triticale OR rye OR fonio OR teff OR “Wild rice” OR spelt OR einkorn OR emmer OR kamut OR “Canary grass” OR quinoa OR amaranth OR buckwheat OR kaniwa OR pitseed OR goosefoot) | | | | | | |
| --- | --- | --- | --- | --- | --- | --- |
| Scopus | #Hits | Web of science | #Hits | Science direct | #Hits | Total articles used |
| Articles | 642 |  | 476 |  | 215 | 1 333 |
| English | 476 |  | 347 |  | 132 | 955 |
| Duplicates | | | | | | 692 |
| Title and abstract screening   - A total of 379 articles reporting on intercrop systems of non-food crops trees, shrubs, cotton, herbs and spices (mint, rosemary and turmeric), alfalfa, silage systems, vegetables were removed. These constituted 33% of the sourced articles, - 150 articles reported on Intercrop systems for cereal-cereal and legume-legume. These constituted 14% of the sourced articles - No mention of intercropping, multicropping and relay cropping (55). These articles had no mention of any multicrop systems or even suggested its application - No mention of water use, water productivity, water use efficiency (54) | | | | | | 54 |
| Screening full text for eligibility   - Measured plant parameters to include yield and water used (evapotranspiration, rainfall received and/ or water applied), - Articles reported yield per unit area of land (kg ha^−1^, t ha^−1^, and g m^-2^), and - The experiments had to have sole crop treatments for both intercrop component species, to allow yield and nutritional yield comparisons between sole crops and intercrops to be compared. | | | | | | 9 |

**Proximate and nutrient composition**

ALL ((“proximate composition” or “nutrient composition”or “nutrient yield”) AND (cereal OR legume) AND (“maize”OR “sorghum” OR “pearl millet” OR “wheat” OR “dry bean” OR “soybean” OR “cowpea” OR “groundnut” OR “pea” OR “chickpea”)

Table 2 References identified through database search and screening procedure

| **Crop type** | **scientific name** | **References** |
| --- | --- | --- |
| **Cereals** | | |
| Maize | *Zea mays L.* | Chandra et al. (2016); Shahidi and Chandrasekara (2013); Okonkwo and Agharandu (2017) Abdulrahman and Omoniyi. (2016); United States Department of Agriculture Food Composition |
| Pearl millet | *Pennisetum glaucum* | Ravindran (1991) ; Osman (2011) ;Chandra et al. (2016); Shahidi and Chandrasekara (2013); Okonkwo and Agharandu (2017) Abdulrahman and Omoniyi. (2016); United States Department of Agriculture Food Composition; http://www.fao.org/infoods/infoods/tables-and-databases/ faoinfoods-databases/en/ |
| Sorghum | *Sorghum bicolor* | Chandra et al. (2016); Shahidi and Chandrasekara (2013); Okonkwo and Agharandu (2017) Abdulrahman and Omoniyi. (2016) Jimoh and Abdullahi (2017); United States Department of Agriculture Food Composition |
| Rice | *Orea Sativa* | Chandra et al. (2016); Shahidi and Chandrasekara (2013); Okonkwo and Agharandu (2017); Abdulrahman and Omoniyi (2016); Lansakara et al. (2016); United States Department of Agriculture Food Composition |
| Wheat | *Triticum aestivum* | Chandra et al. (2016); Shahidi and Chandrasekara (2013); Okonkwo and Agharandu (2017); Abdulrahman and Omoniyi (2016); Lansakara et al. (2016); United States Department of Agriculture Food Composition |
| **Legumes** | | |
| Bambara groundnut | *Vigna subterranea (L.) verdc.* | Chibarabada et al. (2017); Kamboj and Nanda (2018); United States Department of Agriculture Food Composition; http://www.fao.org/infoods/infoods/tables-and-databases/ faoinfoods-databases/en/ |
| Cow pea | *Vigna unguiculata* | Chibarabada et al. (2017); Kamboj and Nanda (2018); International Journal of Innovative Research in Science, Engineering and Technology; United States Department of Agriculture Food Composition |
| Chickpea | *Cicer arietinum* | Qayyum et al. (2012) ; Kabuo et al. (2015); Paul et al. (2011) |
| Dry beans | *Phaseolus vulgaris* | Qayyum et al. (2012) ; Chibarabada et al. (2017); Kamboj and Nanda (2018) |
| Mung bean/ green gram | *Vigna radiate* | Paul et al. (2011) ; Kamboj and Nanda (2018) ; Bindu et al. (2017) ; http ://www.fao.org/infoods/infoods/tables-and-databases/ faoinfoods-databases/en/ |
| Navy bean | *Phaseolus vulgaris* | United States Department of Agriculture Food Composition; http://www.fao.org/infoods/infoods/tables-and-databases/ faoinfoods-databases/en/ |
| Pea | *Pisum sativum* | United States Department of Agriculture Food Composition; http://www.fao.org/infoods/infoods/tables-and-databases/ faoinfoods-databases/en/ |
| Pigeon pea | *Cajanus cajan* | United States Department of Agriculture Food Composition; http://www.fao.org/infoods/infoods/tables-and-databases/ faoinfoods-databases/en/ |
| Soybean | *Glycine max* | United States Department of Agriculture Food Composition; http://www.fao.org/infoods/infoods/tables-and-databases/ faoinfoods-databases/en/ |

References

Okonkwo, C., & Agharandu, U. (2017). Proximate and Vitamin Composition of Selected Cereals commonly used for weaning Babies Food Preparation in South-Eastern Nigeria. Journal of Biology , Agriculture and Healthcare, 7, 22.

Abdulrahman, W. F., & Omoniyi, A. O. (2016). Proximate analysis and mineral compositions of different cereals available in wagwalada market, FCT, Abuja, Nigeria. Journal of Advances in Food Science & Technology, 3(2), 50-55.

Bindu, B. M., Ashwini, M., Vijaykumar, A. G., & Kasturiba, B. (2017). Nutrition composition and antinutritional factors of green gram varieties. Environment and Ecology, 35(3), 1699-1703.

Chandra, D., Chandra, S., & Sharma, A. K. (2016). Review of Finger millet (Eleusine coracana (L.) Gaertn): a power house of health benefiting nutrients. Food Science and Human Wellness, 5(3), 149-155.

Chibarabada, T.P., Modi, A.T., Mabhaudhi, T., 2017. Nutrient content and nutritional water productivity of selected grain legumes in response to production environment. Int. J. Environ. Res. Public Health 14, 1300. Doi:10.3390/ijerph14111300

http://www.fao.org/infoods/infoods/tables-and-databases/ faoinfoods-databases/en/

International Journal of Innovative Research in Science, Engineering and Technology. Abdulrahman, W. F., & Omoniyi, A. O. (2016). Proximate analysis and mineral compositions of different cereals available in wagwalada market, FCT, Abuja, Nigeria. Journal of Advances in Food Science & Technology, 3(2), 50-55.

International Journal of Innovative Research in Science, Engineering and Technology

Iqbal, A., Khalil, I.A., Ateeq, N., Sayyar Khan, M., 2006. Nutritional quality of important food legumes. Food Chem. 97, 331–335. Doi:10.1016/j.foodchem.2005.05.011

Jimoh, W. L. O., & Abdullahi, M. S. (2017). Proximate analysis of selected sorghum cultivars. Bayero Journal of Pure and Applied Sciences, 10(1), 285-288.

Kabuo, N. O., Dialoke, S. A., Omeire, G. C., Bedi, E. N., Peter-Ikechukwu, A. I., & Irekpita, T. E. (2015). Comparison of proximate composition of some cultivars of chickpea (Cicer arietinum L.) cultivated in Owerri, Imo State, Nigeria. Food Science and Quality Management, 37, 103-109.

Kamboj, R., & Nanda, V. (2018). Proximate composition, nutritional profile and health benefits of legumes-A review. Legume Research: An International Journal, 41(3).

Lansakara, L. H. M. P. R., Liyanage, R., Perera, K. A., Wijewardana, I., Jayawardena, B. C., & Vidanarachchi, J. K. (2016). Nutritional composition and health related functional properties of Eleusine coracana (Finger Millet). Procedia food science, 6, 344-347.

Mbanyele, V., Mtambanengwe, F., Nezomba, H., Groot, J. C. J., & Mapfumo, P. (2021). Comparative short-term performance of soil water management options for increased productivity of maize-cowpea intercropping in semi-arid Zimbabwe. Journal of Agriculture and Food Research, 5, 100189. https://doi.org/10.1016/J.JAFR.2021.100189

Okonkwo, C., & Agharandu, U. (2017). Proximate and Vitamin Composition of Selected Cereals commonly used for weaning Babies Food Preparation in South-Eastern Nigeria. Journal of Biology, Agriculture and Healthcare, 7, 22.

Osman, M. A. (2011). Effect of traditional fermentation process on the nutrient and antinutrient contents of pearl millet during preparation of Lohoh. Journal of the Saudi Society of Agricultural Sciences, 10(1), 1-6.

Paul, T., Mozumder, N. R., Sayed, M. A., Akhtaruzzaman, M., & Akhtaruzzaman, M. S. M. (2011). Proximate compositions, mineral contents and determination of protease activity from green gram (Vigna radiata L. Wilczek). Bangladesh Res. J, 5, 207-213.

Qayyum, M. M. N., Butt, M. S., Anjum, F. M., & Nawaz, H. (2012). Composition analysis of some selected legumes for protein isolates recovery. The journal of Animal and plant sciences, 22(4), 1156-1162.

Ravindran, G. (1991). Studies on millets: Proximate composition, mineral composition, and phytate and oxalate contents. Food Chemistry, 39(1), 99-107.

Shahidi, F., & Chandrasekara, A. (2013). Millet grain phenolics and their role in disease risk reduction and health promotion: A review. Journal of Functional Foods, 5(2), 570-581.

United States Department of Agriculture Food Composition. https://ndb.nal. Usda.gov/ndb

Table 3: Description of selected studies for the analysis.

| **Data source** | **Location** | **Geographic coordinates** | **Elevation** | **Rainfall^1^** | **Cropping system** | | **Intercrop design** | **Experimental units^2^** |
| --- | --- | --- | --- | --- | --- | --- | --- | --- |
|  |  |  |  |  | *Cereal* | *Legume* |  |  |
| Chen et al., 2018 | China | 37°96′N; 102°64′E | 1506 | 168^448^ | Maize (*Zea mays*) | Pea (*Pisum sativum*) | Additive | 15 |
| Chimonyo et al., 2016 | South Africa | 28°01’S; 28°99’E | 998 | 490^102^ | Sorghum (*Sorghum bicolor*) | Cowpea (*Vigna unguiculata*) | Additive | 6 |
| Feng et al., 2016 | China | 42°8’N; 121°46’E | 270 | 481 | Foxtail millet (*Setaria italica* L. Beauv.) | Groundnut (*Arachis hypogea*) | Additive | 8 |
| Mao et al., 2012 | China | 38°37′N; 102◦40′ E | 1504 | 150^438^ | Maize (*Zea mays*) | Pea (*Pisum sativum*) | Additive | 12 |
| Miriti et al., 2012 | Kenya | 1°50’S; 37°40’E | 1155 | 314 | Maize (*Zea mays*) | Cowpea (*Vigna unguiculata*) | Additive | 20 |
| Tsubo et al., 2003 | South Africa | 29°01’S; 26°09’E | 1354 | 601^108^ | Maize (*Zea mays*) | Dry beans (*Phaseolus vulgaris*) | Additive | 7 |
| Jahansooz et al., 2007 | Australia | 34°32’S, 138°41’E | 113 | 420^131^ | Wheat (*Triticum aestivum*) | Chickpea (*Cicer arietinum*) | Additive | 6 |
| Choudhary and Kumar, 2016 | India | 27°95’S, 94°76’E | 660 | 858 | Maize (*Zea mays*) | Soybean (*Glycine max*)/ Groundnut (*Arachis hypogea*) | Additive | 6 |
| Mbanyele et al., 2021 | Zimbabwe | 18°31’S; 31°37’E | 1 409 | 850 | Maize (*Zea mays*) | Cowpea (*Vigna unguiculata*) | Additive | 12 |

^1^Rainfall presented represents the average seasonal precipitation received. The number presented as a superscript next to rainfall represents the average irrigation applied in the season. ^2^Experimental unit was defined as a combination of site, year and treatment (intercrop and management factors).

Table 4: Average bioavailable nutrient composition (per 100 g at 12 % moisture) of cereals and legumes pooled from the literature on the nutrition content of identified crops.

| Crop type | Scientific name | Nutrients (bioavailability) | | | | | |
| --- | --- | --- | --- | --- | --- | --- | --- |
|  |  | Protein (g) | Carbohydrate (g) | Fibre (g) | Iron (mg) | Zinc (mg) | Calcium (mg) |
| Maize | *Zea mays* | 1.8 | 15.9 | 1.6 | 0.3 | 0.12 | 1.3 |
| Fox millet | *Setaria italica* | 2.3 | 15.2 | 0.3 | 0.4 | 1.4 | 2.0 |
| Sorghum | *Sorghum bicolor* | 5.2 | 54.6 | 5.2 | 1.4 | - | - |
| Wheat | *Triticum aestivum* | 2.0 | 10.2 | 1.7 | 0.7 | 0.4 | 6.5 |
| Cowpea | *Vigna unguiculata* | 2.9 | 7.1 | 1.3 | 1.1 | 0.7 | 9.1 |
| Dry beans | *Phaseolus vulgaris* | 2.8 | 6.2 | 2.6 | 0.8 | 0.4 | 13.9 |
| Groundnut | *Arachis hypogaea* | 4.7 | 7.4 | 3.1 | 0.4 | 0.6 | 19.3 |
| Pea | *Pisum sativum* | 2.9 | 5.3 | 2.1 | 0.5 | 0.4 | 4.9 |
| Chickpea | *Cicer arietinum* | 3.1 | 7.3 | 2.3 | 1.0 | 0.5 | 17.2 |
| Soybean | *Glycine max* | 5.8 | 1.7 | 1.8 | 1.8 | 0.4 | 35.7 |

Table 5: Population and fertiliser rates used and total water applied in experiments in selected studies and water rates of intercrop systems used in the study

| **Cropping systems** | | **Plant population** | | | | **N (kg ha^-1^)** | **P_2_O_5_ (kg ha^-1^)** | **K (kg ha^-1^)** |
| --- | --- | --- | --- | --- | --- | --- | --- | --- |
| Cereal | Legume | Cereal - sole | Cereal - Intercrop | Legume - sole | Legume - Intercrop |  |  |  |
| Maize | Cowpea | 3.7 | 3.7 | 3.7 | 3.7 | 20 | 40 | - |
|  | Dry bean | 2.2 – 6.7 (5)^1^ | 2.2 – 6.7 (5) | 2.1 – 10 (6.6) | 2.1 – 10 (6.6) | 172 – 254 (226) | 47 – 96 (70) | 32 – 48 (37) |
|  | Pea | 6.3 – 8.3 (7) | 3.1 – 8.3 (6.5) | 13 – 24 (17) | 10 – 24 (16.5) | 240 – 300 (270) | 90 | - |
|  | Groundnut | 5.5 | 5.5 | 33 | 11.1 -43 (22.2) | 80/25^2^ | 60/60 | 40/40 |
|  | Soybean | 5.5 | 5.5 | 33 | 11.1 -43 (22.2) | 80/25 | 60/60 | 40/40 |
| Fox millet | Groundnut | 48 | 48 | 24 | 24 | 15 | 15 | 15 |
| Sorghum | Cowpea | 2.6 | 1.3 – 3.9 (2.6) | 0.76 | 0.65 – 1.3 (0.98) | 85 | 15 | 15 |
| Wheat | Chickpea | 155 | 40 | 155 | 40 | 50 | 20 | - |

^1^Number in brackets represent the mean,^2^Number after the forward-slash represents fertiliser applied to the legume system.

Table 6: Daily Dietary Reference Intake (DRI) for an average family of four comprising of an adult male and female, adolescent female and a child

| **Age group** | **Protein requirements (g kg^-1^ d^-1^)** | **Protein needed (g d^-1^)** | **CHO^2^ (g d^-1^)** | **Fe^3^ (mg d^-1^)** | **Zn^4^ (mg d^-1^)** | **Ca^5^ (mg d^-1^)** |
| --- | --- | --- | --- | --- | --- | --- |
| Adult male | 0.66 | 52.8 | 100 | 6 | 9.4 | 1000 |
| Adult Female | 0.66 | 49.5 | 100 | 8.1 | 6.8 | 1000 |
| Adolescent^1^ Female | 0.71 | 39.1 | 100 | 7.9 | 7.3 | 1300 |
| Child (4-8 years) | 0.76 | 19 | 100 | 4.1 | 4.0 | 800 |
| Total | 2.79 | 160.4 | 400 | 26.1 | 27.5 | 4100 |

^1^According to Sawyer et al. (2018) 10–24 years corresponds more closely to adolescent growth and popular understandings of this life phase and would facilitate extended investments across a broader range of settings; ^2^CHO – Carbohydrates; ^3^Fe – Iron; ^4^Zn – Zinc; ^5^Ca – Calcium.

Table 7 Nutritional yield of intercrop systems relative to corresponding sole cereal system

Protein NC ranged from 1.78 to 5.2 g 100 g^-1^; maize and sorghum had the lowest and highest protein concentration, respectively. For carbohydrates concentration, values ranged from 5.32 to 54.6 g 100 g-1; the lowest and highest carbohydrates concentration were obtained from pea and sorghum, respectively. For micronutrients, maize and sorghum indicated the lowest (0.26 mg 100 g^-1^) and highest (1.4 mg 100 g^-1^) Fe concentration, respectively. Zinc concentration ranged from 0.00 to 1.4 mg 100 g^-1^; the lowest and highest Zn concentration were attained from sorghum and pearl millet, respectively. Similarly, sorghum and groundnut indicated the lowest (0.00 mg 100 g-1), and highest (19.25 mg 100 g^-1^) Ca concentration, respectively. Generally, our results suggest that cereals are higher in carbohydrates concentrations, whereas legumes are nutrient-dense in micronutrients, except for the higher Fe nutrient concentration, which was observed for sorghum.

| **Cropping system** | | **Cereal crop ^1^** | | | | | | **Intercropping** | | | | | |
| --- | --- | --- | --- | --- | --- | --- | --- | --- | --- | --- | --- | --- | --- |
|  |  | -------------------------------------------------------------------------------------- (kg ha^-1^) ----------------------------------------------------------------------- | | | | | | | | | | | |
| Cereal | Legume | CHO | Protein | Fibre | Iron | Zinc | Calcium | CHO | Protein | Fibre | Iron | Zinc | Calcium |
| Maize | Cowpea | 57 - 1641  (767)^2^ | 6 - 183  (86) | 6 - 161  (75) | 0.001 – 0.027  (0.013) | 0.001 – 0.022  (0.001) | 0.005 – 0.134  (0.063) | 30 – 267 (142) | 5 – 42 (20) | 3 – 29 (15) | 0.001 – 1.0 (0.4) | 0.001 – 0.6 (0.3) | 0.009 – 0.072 (0.030) |
|  | Dry bean | 398 -1641  (1124) | 45 - 183  (126) | 39 - 161  (110) | 0.007 – 0.0023  (0.018) | 0.004 – 0.006  (0.005) | 0.033 -  0.134  (0.092) | 43 – 1527 (1032) | 48 – 201 (138) | 11 – 179 (123) | 0.005 – 0.036 (0.025) | 0.005 – 0.019 (0.013) | 0.000 – 0.326 (0.233) |
|  | Pea | 1073 - 2772  (1767) | 120 - 310  (184) | 105 - 271  (161) | 0.018 – 0.045  (0.027) | 0.010 – 0.026  (0.017) | 0.088 – 0.226  (0.134) | 1060 – 2083 (1505) | 142 – 259 (167) | 120 – 222 (159) | 0.021 – 0.038 (0.020) | 0.013 – 0.023 (0.018) | 0.129 – 0.220 (0.145) |
|  | Groundnut | 636 | 71 | 62 | 0.010 | 0.006 | 0.052 | 494 – 651 (578) | 71 – 82 (76) | 47 – 70 (57) | 0.018 – 0.025 (0.021) | 0.044 – 0.063 (0.053) | 0.309 – 457 (375) |
|  | Soybean | 636 | 71 | 62 | 0.010 | 0.006 | 0.052 | 396 - 636  (545) | 92 - 142  (116) | 66 - 75  (69) | 0.017 – 0.037  (0.026) | 0.007 – 0.010  (0.009) | 0.203 – 0.649  (0.394) |
| Fox millet | Groundnut | 727 - 818  (773) | 109 - 123  (116) | 13 - 14  (13) | 0.019 – 0.021  (0.020) | 0.067 – 0.076  (0.071) | 0.094 – 0.105  (0.100) | 556 - 794  (671) | 104 - 148  (124) | 47 - 90  (64) | 0.048 – 0.065  (0.054) | 0.078 – 0.096  (0.087) | 0.409 – 0.562  (0.455) |
| Sorghum | Cowpea | 410 - 650  (511) | 39 - 62  (49) | 39 - 62  (49) | 0.011 – 0.017  (0.013) | 0.016 – 0.025  (0.020) | 0.011 – 0.018  (0.014) | 458 - 765  (568) | 39 - 75  (52) | 39 - 73  (52) | 0.011 – 0.020  (0.014) | 0.016 – 0.030  (0.021) | 0.011 – 0.029  (0.018) |
| Wheat | Chickpea | 154 - 445 (303) | 31 - 88  (60) | 26 - 74  (50) | 0.010 – 0.028  (0.019) | 0.006 – 0.016  (0.011) | 0.098 – 0.284  (0.193) | 123 - 327  (230) | 27 – 77  (52) | 22 - 62  (42) | 0.009 – 0.025  (0.017) | 0.005 – 0.014  (0.009) | 0.099 – 0.300  (0.194) |

^1^All values are present as X10^3^; ^2^Number in brackets represents the mean

Table 8 Nutritional water productivity (g m^3^) of intercrop systems relative to corresponding sole cereal system

| **Cropping system** | | **Cereal crop** | | | | | | **Cereal-Legume intercrop** | | | | | |
| --- | --- | --- | --- | --- | --- | --- | --- | --- | --- | --- | --- | --- | --- |
| **Cereal** | **Legume** | -----------------------gm^-3^--------------- | | | ----------------X10^3^ gm^-3^--------- | | | -----------------------gm^-3^------------- | | | ----------------X10^3^ gm^-3^--------- | | |
|  |  | CHO | Protein | Fibre | Iron | Zinc | Calcium | CHO | Protein | Fibre | Iron | Zinc | Calcium |
| **Maize** | **Cowpea** | 258.32 - 1153.55  (761.18) | 28.86 - 128.90  (85.05) | 25.30 - 112.97  (74.54) | 4.21 - 18.83  (12.42) | 2.43 - 10.86  (7.16) | 21.08 - 94.14  (62.11) | 238.70 – 2304.00  (1284.65) | 47.96 – 463  (258.16) | 29.22 – 282  (157.24) | 13.78 – 133.00  (74.16) | 8.29 – 80.00  (44.61) | 107.75 – 1040.00  (579.88) |
|  | **Drybean** | 1378.39 - 2129.01  (1722.40) | 154.02 - 237.89  (192.46) | 134.98 - 208.49  (168.67) | 22.50 - 34.75  (28.11) | 13.00 - 20.05  (16.22) | 112.49 - 173.74  (140.56) | 1745.19 - 2992.42  (2410.27) | 357.23 - 612.54  (493.38) | 324.91 - 557.11  (448.73) | 84.38 - 144.69  (116.54) | 40.22 - 68.96  (55.55) | 1198.69 - 2055.35  (1655.50) |
|  | **Groundnut** | 731.95 | 81.79 | 71.67 | 11.95 | 6.89 | 59.73 | 1036.45 - 1235.42  (1128.79) | 178.26 - 212.49  (194.15) | 168.36 - 200.68  (183.36) | 28.00 - 33.36  (30.48) | 20.24 - 24.12  (22.04) | 704.03 - 839.18  (766.75) |
|  | **Pea** | 1736.37 - 4888.57  (3004.43) | 194.02 - 546.24  (335.71) | 170.04 - 478.73  (294.22) | 28.34 - 79.79  (49.04) | 16.35 - 46.03  (28.29) | 141.70 - 398.94  (245.18) | 3214.51 - 5047.27  (3971.91) | 707.95 - 1111.59  (874.76) | 549.11 - 862.19  (678.50) | 108.92 - 171.01  (134.58) | 75.64 - 118.76  (93.46) | 937.88 - 1472.61  (1158.86) |
|  | **Soybean** | 731.95 | 81.79 | 71.67 | 11.95 | 6.89 | 59.73 | 828.72 - 973.85  (896.98) | 357.18 - 419.73  (386.60) | 157.65 - 185.26  (170.64) | 96.47 - 113.37  (104.42) | 25.88 - 30.42  (28.01) | 1741.20 - 2046.14  (1884.63) |
| **Millet** | **Groundnut** | 1961.87 - 2196.98  (2105.70) | 293.96 - 329.18  (315.51) | 33.67 - 37.70  (36.14) | 50.50 - 56.56  (54.21) | 181.29 - 203.02  (194.59) | 252.52 - 282.78  (271.03) | 2927.51 - 4503.20  (3421.40) | 581.98 - 895.22  (680.17) | 328.07 - 504.65  (383.42) | 98.05 - 150.82  (114.59) | 216.20 - 332.57  (252.68) | 2136.87 - 3287.01  (2497.37) |
| **Sorghum** | **Cowpea** | 1115.05 - 3214.38  (1892.98) | 106.20 - 306.13  (180.28) | 106.20 - 306.13  (180.28) | 28.59 - 82.42  (48.54) | 42.89 - 123.63  (72.81) | 30.63 - 88.31  (52.00) | 1319.26 - 3117.89  (2308.48) | 125.64 - 406.73  (274.37) | 125.64 - 326.39  (234.48) | 33.83 - 124.80  (81.46) | 50.74 - 138.94  (98.23) | 36.24 - 535.56  (286.83) |
| **Wheat** | **Chickpea** | 711.66 - 1482.92  (1218.23) | 140.94 - 293.68  (241.26) | 117.91 - 245.70  (201.84) | 45.35 - 94.50  (77.63) | 25.82 - 53.79  (44.19) | 453.51 – 945.00  (776.32) | 974.57 - 2108.09  (1679.33) | 286.57 - 619.88  (493.81) | 223.01 - 482.4  (384.29) | 93.11 - 201.40  (160.44) | 50.74 - 109.75  (87.43) | 1318.56 - 2852.19  (2272.10) |

^1^Number in brackets represent the mean

Table 9 Nutritional contribution for a family of four for a year

| Cropping system | | Cereal (%) | | | | | Intercrop (%) | | | | |
| --- | --- | --- | --- | --- | --- | --- | --- | --- | --- | --- | --- |
| Cereal | Legume | CHO | Protein | Iron | Zinc | Calcium | CHO | Protein | Iron | Zinc | Calcium |
| Maize | Cowpea | 39 - 1046  (485)^1^ | 11 - 344  (160) | 10 - 373  (174) | 5 - 188  (88) | 0 - 22  (10) | 20 - 183  (97) | 8 - 72  (35) | 13 - 105  (46) | 7 - 59  (26) | 1 - 5  (2) |
|  | Dry bean | 273 - 1124  (770) | 76 - 313  (215) | 68 - 281  (193) | 37 -  154  (105) | 2 - 9  (6) | 180 - 1046  (707) | 82 - 344  (236) | 112 - 373  (260) | 52 - 188  (130) | 9 - 22  (16) |
|  | Pea | 735 - 1899  (1128) | 205 - 529  (314) | 184 - 475  (282) | 101 - 260  (154) | 6 - 15  (9) | 726 - 1427  (1031) | 243 - 442  (342) | 221 - 401  (313) | 129 - 229  (183) | 9 - 15  (12) |
|  | Groundnut | 436 | 122 | 109 | 60 | 3 | 338 - 446  (396) | 121 -  141  (130) | 111 - 130  (120) | 64 -  78  (73) | 7 - 15  (11) |
|  | Soybean | 436 | 122 | 109 | 60 | 3 | 271 - 436  (374) | 157 - 242  (199) | 184 - 389  (275) | 73 - 103  (89) | 14 - 43  (26) |
| Fox Millet | Groundnut | 498 -560  (529) | 186 - 209  (198) | 197 - 221  (209) | 669 - 753  (711) | 6 - 7  (7) | 381 - 544  (459) | 177 - 252  (212) | 184 - 262  (220) | 434 - 624  (529) | 21 - 31  (25) |
| Sorghum | Cowpea | 280 - 445  (350) | 67 - 106  (83) | 110 - 175  (138) | 157 - 249  (196) | 1 - 1  (1) | 280 - 524  (371) | 67 - 128  (89) | 110 - 214  (149) | 157 - 297  (209) | 1 - 2  (1) |
| Wheat | Chickpea | 84 - 224  (158) | 46 - 132  (89) | 92 - 261  (176) | 49 - 138  (94) | 7 - 20  (13) | 106 - 305  (208) | 52 - 151  (103) | 103 - 298  (203) | 56 - 161  (110) | 7 - 19  (13) |

^1^Number in brackets represents the mean
