# Supplementary material for: Can cereal-legume intercrop systems contribute to household nutrition in semi-arid environments: A systematic review and meta-analysis
Source: Front Nutr. 2023 Jan 26;10:1060246. doi: 10.3389/fnut.2023.1060246 (PMC9923432; doi:10.3389/fnut.2023.1060246)
Supplement: Supplementary file 3 [file Table_2.docx]

**Supplementary information 2 -** Glossary of terms and definition

**Experiment** was defined at a field trial conducted with a clear set of factors

**Proximate composition** of foods includes moisture, ash, lipid, protein and carbohydrate contents. These food components may be of interest in the food industry for product development, quality control (QC) or regulatory purposes.

**Land Equivalent Ratio (LER)** is defined as the relative land area required as a sole crops to produce the same yields as intercropping (Hossein Pour et al., 2016)

**Dietary Reference Intakes** **(DRI)** is the general term for a set of reference values used to plan and assess nutrient intakes of healthy people. These values, which vary by age and sex. (https://ods.od.nih.gov/Health_Information/Dietary_Reference_Intakes.aspx)
