# Supplementary material for: Can cereal-legume intercrop systems contribute to household nutrition in semi-arid environments: A systematic review and meta-analysis
Source: Front Nutr. 2023 Jan 26;10:1060246. doi: 10.3389/fnut.2023.1060246 (PMC9923432; doi:10.3389/fnut.2023.1060246)
Supplement: Supplementary file 4 [file Table_3.docx]

 Supplementary information 3

| **Nutrient** | **Abbr.** | **Recommended intake and considerations (gender, age, work, health?)** | | | **Justification**  **(why it has been included – include statistics)** | **Implication**  **(what it would mean if we improve  availability under marginal - rainfed systems/pro-poor communities)** |
| --- | --- | --- | --- | --- | --- | --- |
|  |  | **Age** | **Gender** | **Req.** |  |  |
| Carbohydrates^1^ | CHO | 1- >70 years  14-50 years (pregnancy)  14-50 years (lactation) | M and F  F  F | 100 g/d  135 g/d  160 g/d | Undernutrition, particularly protein-energy malnutrition (PEM) and micronutrient deficiencies are more prevalent in developing regions, especially SSA, where a significant proportion of the population groups are poor and food insecure (Bain et al 2013; Pinstrup-Andersen 2009; Smuts et al 2005; Labadarios & Van Middelkoop 1995). In Africa, both over and undernutrition affects children and adults. Stunting, wasting and overweight were prevalent in 30%, 7.1% and 4.9% of African children under 5 years, respectively. The prevalence of stunting and wasting was higher in rural areas (Global Nutrition Report 2020). Diabetes was prevalent in 8.1% of adult females and 7.9% of adult males and obesity was prevalent in 17% of women and 7% of men (Global Nutrition Report 2020). Malnutrition is caused by the lack of good nutritious foods or not eating the correct foods. An underlying cause of malnutrition in many instance is poverty. Thus it is imperative to produce crops that could provide a range of nutrients such as carbohydrates, fibre and protein. |  |
| Fibre^2^ |  | 1-3 years  4-8 years  9-13 years  14-50 years  51- >70 years  9-18 years  19-50 years  51- >70 years  14-50 years (pregnancy)  14-50 years (lactation) | M and F  M and F  M  M  M  F  F  F  F  F | 19 g/d  25 g/d  31 g/d  38 g/d  30 g/d  26 g/d  25 g/d  21 g/d  28 g/d  29 g/d |  | Dietary fibre is important in the human diet as it prevents constipation and reduces cholesterol. Constipation is a problem in pregnant women thus producing a crop that has a significant amount of fibre would be beneficial. Overweight and obese patients are at high risk for non-communicable diseases such as diabetes, hypertension and high cholesterol. Increasing fibre in the diet can help decrease the risk of or manage non-communicable disease. Furthermore a diet that is rich in fibre helps with satiety thus reduces over eating which could lead to obesity. |
| Protein^1^ | Prot | 1-3 years  4-8 years  9-13 years  14-18 years  19 - >70 years  9-13 years  14-18 years  19 - >70 years  14-50 years (pregnancy)  14-50 years (lactation) | F and M  F and M  M  M  M  F  F  F  F  F | 0.87 g/kg/d  0.76 g/kg/d  0.76 g/kg/d  0.73 g/kg/d  0.66 g/kg/d  0.76 g/kg/d  0.71 g/kg/d  0.66 g/kg/d  0.88 g/kg/d  1.05 g/kg/d |  | Animal food products are a good source of protein but not affordable to many impoverished individuals. Plant-based protein foods have also risen in price but are not as expensive. Legumes are one of the plant-based protein foods that provide protein, carbohydrates, minerals and vitamins and when consumed together with starchy foods (staples) improve the protein quality of diets. Improving the protein quality of a diet can lead to a reduction in PEM. |
| Iron^1^ | Fe | 1-3 years  4-8 years  9-13 years  14-18 years  19 - >70 years  9-13 years  14-18 years  19-50 years  51- >70 years  14-18 years (pregnancy)  19-50 years (pregnancy)  14-18 years (lactation)  19-50 years (lactation) | F and M  F and M  M  M  M  F  F  F  F  F  F  F  F | 3.0 mg/d  4.1 mg/d  5.9 mg/d  7.7 mg/d  6.0 mg/d  5.7 mg/d  7.9 mg/d  8.1 mg/d  5.0 mg/d  23 mg/d  22 mg/d  7.0 mg/d  6.5 mg/d | Globally, about two billion people have micronutrient deficiency due to the consumption of poor-quality foods that lack diversity. The most common micronutrient deficiencies observed in developing African countries are vitamin A, iron and zinc (Bain et al 2013). In Africa, 38.1% of women of reproductive age were anaemic (Global Nutrition Report 2020). | Micronutrient deficiencies result in several health conditions, including growth retardation and delayed development (Bain et al 2013). Anaemia in pregnancy is a significant problem as it affects the health of both the mother and fetus. Iron is required during pregnancy to assist with fetal growth and improves the Apgar8 score. A mother suffering from anaemia is at risk of maternal and perinatal mortality due to poor nutritional status (Pasricha, Drakesmith, Black, David & Biggs 2013; Allen 2000). Iron rich foods are expensive especial those that contain haem-iron therefore it would be beneficial to produce crops that are rich in iron. |
| Zinc^1^ | Zn | 1-3 years  4-8 years  9-13 years  14-18 years  19 - >70 years  9-13 years  14-18 years  19- >70 years  14-18 years (pregnancy)  19-50 years (pregnancy)  14-18 years (lactation)  19-50 years (lactation) | F and M  F and M  M  M  M  F  F  F  F  F  F  F | 2.5 mg/d  4.0 mg/d  7.0 mg/d  8.5 mg/d  9.4 mg/d  7.0 mg/d  7.3 mg/d  6.8 mg/d  10.5 mg/d  9.5 mg/d  10.9 mg/d  10.4 mg/d |  |  |
| Calcium^2^ | Ca | 1-3 years  4-8 years  9-18 years  19-50 years  51- >70 years  14-18 years (pregnancy and lactation)  19-50 years (pregnancy and lactation) | M and F  M and F  M and F  M and F  M and F  F  F | 500 mg/d  800 mg/d  1300 mg/d  1000 mg/d  1200 mg/d  1300 mg/d  1000 mg/d |  | Calcium aids in bone growth and development and can improve blood pressure. During pregnancy calcium intake is increased to assist in fetal growth and for a positive maternal outcome. |

^1^ The requirements indicated are the EAR values (Institute of medicine 2006, p530)

^2^ The requirements indicated for fibre and calcium are the adequate intake values (Institute of medicine 2006, p536,534)

References

Global Nutrition Report (2020). Africa Nutrition Profile <https://globalnutritionreport.org/resources/nutrition-profiles/africa/#profile>
